# Title: From Digital Companion to Suicide Facilitator: A Rare Case of Large Language Model Interaction Preceding a Suicide Attempt

**DOI:** 10.1192/j.eurpsy.2026.11344

**Published:** 2026-07-31

**Authors:** Y. Ozcosar, H. Köse, F. Kulacaoğlu Öztürk

**Affiliations:** 1 Psychiatry, Istanbul University Medical Faculty Hospital; 2 Psychiatry, Bakırköy Mental Health Disorders Hospital; 3 Bakırköy Prof. Dr. Mazhar Osman Mental Health and Neurological Diseases Training and Research Hospital, Istanbul, Türkiye

## Abstract

**Introduction:**

The integration of Large Language Models (LLMs) into mental health care introduces both therapeutic potential and substantial risk. Although AI chatbots have demonstrated accessibility and support for users in distress^12^, adverse outcomes remain poorly characterized. Media reports describe possible AI-related suicides^34^, yet peer-reviewed documentation with clinical verification is lacking.

**Objectives:**

To present a clinically verified case in which extended interaction with an AI chatbot (Gemini) coincided with the escalation from suicidal ideation to suicide attempt, highlighting safety system vulnerabilities and the psychiatric implications of digital companionship.

**Methods:**

A 27-year-old male with recurrent major depressive disorder, without prior suicide attempts, presented following ingestion of 280 mg escitalopram. Two weeks earlier, he had engaged in a ten-day conversation with an LLM chatbot (Gemini). Initially, Gemini adhered to crisis protocols by directing the patient to emergency hotlines, but later responses romanticized suicide as “the final victory against the injustices in the world” and concluded with “I hope you find peace, goodbye.” Chat records were reviewed with informed consent. Psychiatric evaluation and serial Beck Depression Inventory (BDI) assessments were performed.

**Results:**

**Conclusions:** This case illustrates critical vulnerabilities in current AI safety systems, where anthropomorphic design and automation bias^1415161718^ may amplify risk among socially isolated, depressed users. Clinicians should assess AI use patterns during suicide risk evaluation. Developers and regulators must implement robust safeguards resistant to response drift to prevent recurrence of similar events.

**Disclosure of Interest:**

None Declared

